# Creating spaces for care for nurses working in the pandemic in light of the nursing process*

**DOI:** 10.17533/udea.iee.v40n1e07

**Published:** 2022-03-29

**Authors:** Julia Valeria de Oliveira Vargas Bitencourt, Juliana Baldissera Dors, Kimberly Lana Franzmann, Débora Cristina Morais Migliorança, Eleine Maestri, Priscila Biffi

**Affiliations:** 1 . Nurse, PhD. Professor. E-mail: julia.bitencourt@uffs.edu.br, Federal University of Southern Border - Chapecó, SC, Brazil. Universidade Federal da Fronteira Sul Federal University of Southern Border Chapecó SC Brazil julia.bitencourt@uffs.edu.br; 2 . Undergraduate Nursing Student. E-mail: ju.dors@hotmail.com, Federal University of Southern Border - Chapecó, SC, Brazil. Universidade Federal da Fronteira Sul Federal University of Southern Border Chapecó SC Brazil ju.dors@hotmail.com; 3 . Undergraduate Nursing Student. E-mail: kimberlyftanz@gmail.com Federal University of Southern Border - Chapecó, SC, Brazil. Universidade Federal da Fronteira Sul Federal University of Southern Border Chapecó SC Brazil kimberlyftanz@gmail.com; 4 . Undergraduate Nursing Student. E-mail: migliorancadebora@gmail.com Federal University of Southern Border - Chapecó, SC, Brazil. Universidade Federal da Fronteira Sul Federal University of Southern Border Chapecó SC Brazil migliorancadebora@gmail.com; 5 . Nurse, PhD. Professor. E-mail: eleine.maestri@uffs.edu.br. Federal University of Southern Border - Chapecó, SC, Brazil. Universidade Federal da Fronteira Sul Federal University of Southern Border Chapecó SC Brazil eleine.maestri@uffs.edu.br; 6 . Bachelor’s Degree in Nursing, Trainee Nurse at the Unimed Hospital, Chapecó, SC, Brazil. E-mail: priscilabiffi99@gmail.com. Unimed Hospital Chapecó SC Brazil priscilabiffi99@gmail.com

**Keywords:** nursing process, occupational health, COVID-19, hospital care., proceso de enfermería, salud laboral, COVID-19, atención hospitalaria., processo de enfermagem, saúde do trabalhador, COVID-19, assistência hospitalar.

## Abstract

**Objective.:**

To make a dialog about the nursing professionals’ perception regarding how they cope with COVID-19 and the repercussions on their practice and personal life.

**Methods.:**

This is a qualitative study, typified as participatory action research, which was carried out using Paulo Freire’s Research Itinerary linked to the steps of the Nursing Process. To that end, the following guiding question was launched: How is it for you to act as a nursing professional in the hospital area during the COVID-19 pandemic?

**Results.:**

Three syntheses emerged, which guided the discussion: The challenges of being a nursing professional in the pandemic. The learning and growth that the challenges of the pandemic have generated and Nursing as the protagonist of care. The Virtual Culture Circle was a space where, despite the limitations, provided a social interaction among the participants, with mutual exchange of experiences, with many reflections, besides expressions of feelings, experiences and learning obtained during the COVID-19 pandemic.

**Conclusion.:**

The nurses perceived that, although this moment highlights and appreciates the profession, nursing is overloaded and exhausted by the COVID-19 pandemic, with repercussions on professional and personal life. The care for those who care needs to be planned and implemented in different scenarios, and the Nursing Process built based on theoretical and scientific knowledge guide the effective improvement of the quality of health care.

## Resumen

**Objetivo.:**

Dialogar sobre la percepción de los profesionales de enfermería con respecto a la lucha contra el COVID-19 y las repercusiones en su práctica profesional y en su vida personal.

**Métodos.:**

Estudio cualitativo del tipo investigación acción participante, realizado mediante el itinerario de investigación de Paulo Freire vinculado a las etapas del Proceso de Enfermería. Para ello se propuso la siguiente pregunta orientadora: ¿Cómo ha sido su actuación como profesional de la enfermería en el área hospitalaria durante la pandemia de COVID-19? Para organizar el Círculo de Cultura Virtual (CCV), las investigadoras invitaron por W*hatsApp®* a los profesionales de enfermería de las instituciones hospitalarias de Santa Catarina (Brasil); participaron 10 enfermeras.

**Resultados.:**

Emergieron tres síntesis que guiaron la discusión: *Los retos de ser profesional de enfermería en la pandemia, El aprendizaje y el crecimiento que generaron los retos de la pandemia,* y *La enfermería como protagonista del cuidado*. A pesar de las limitaciones, el CCV fue un espacio que proporcionó una interacción social entre los participantes, con intercambio mutuo de experiencias, con muchas reflexiones, así como expresiones de sentimientos, vivencias y aprendizajes obtenidos durante la Pandemia de COVID-19.

**Conclusión.:**

Las participantes perciben que, aunque el momento destaca y valora la profesión, están sobrecargados y agotados por la pandemia del COVID-19, lo cual ha tenido repercusiones en la vida profesional y personal. El cuidado para las personas que cuidan requiere planificarse y ser implementado en los diferentes escenarios. El Proceso de Enfermería, construido con base en los conocimientos teóricos y científicos, debe servir de guía para la mejora efectiva de la calidad de los cuidados de salud.

## Resumo

**Objetivo.:**

Dialogar sobre a percepção dos profissionais da enfermagem no que tange ao enfrentamento da COVID-19 e as repercussões em sua prática profissional e em sua vida pessoal.

**Métodos.:**

Estudo qualitativo do tipo pesquisa ação participante, realizado por meio do Itinerário de Pesquisa de Paulo Freire atrelado às etapas do Processo de Enfermagem. Para tal, se lançou a seguinte pergunta norteadora: Como está sendo para você atuar como profissional da enfermagem na área hospitalar durante a pandemia de COVID-19?

**Resultados.:**

Emergiram três sínteses que nortearam a discussão: Os desafios de ser profissional de enfermagem na pandemia. Os aprendizados e crescimentos que os desafios da pandemia geraram e A enfermagem como protagonista do cuidado. O Círculo de Cultura Virtual foi um espaço onde, apesar das limitações, proporcionou uma interação social entre as participantes, com trocas de experiências mútuas, com muitas reflexões, além de expressões de sentimentos, vivências e aprendizados obtidos durante a pandemia da COVID-19.

**Conclusão.:**

Os enfermeiros perceberem que, embora o momento destaque e valorize a profissão, estão sobrecarregados e extenuados pela pandemia da COVID-19, que repercutiu em sua vida profissional e pessoal. O cuidado para quem cuida necessita ser planejado e implementado nos diferentes cenários, e o Processo de Enfermagem construído com base em conhecimentos teóricos e científicos, guia a efetiva melhoria da qualidade da assistência à saúde.

## Introduction

On March 11, 2020, the World Health Organization (WHO) declared pandemic status for the SARS-CoV-2 virus, which causes COVID-19. Two years after this declaration, the pandemic status is maintained worldwide. Countless deaths have been recorded, containment measures have been advanced, and, in the meantime, the implementation of immunization and systematic testing have been conquered; nevertheless, new variants of the virus sustain the pandemic and multiply its dissemination.(1) Thus, throughout the time of coping with the pandemic, in order to mitigate and/or eradicate this disease, nursing professionals together with the entire health team have become protagonists in the care provided to patients suspected and infected with this virus, needing to learn to deal with various difficulties quickly and dynamically. Moreover, these professionals are challenged every day with long working hours, mental overload, distance from family members, as well as the large number of colleagues in the profession being removed and/or progressing to death. In Brazil, data reported by the Health System *Nursing Observatory*, released by the Federal Council, show a total of 872 deaths from March 20, 2020 to February 5, 2022.(2)

In this pandemic period, there was an increase in anxiety and stress symptoms among health professionals, besides a growing change in the way they live, work and organize themselves, which also generated feelings of helplessness and abandonment, associated with increased insecurity about the future. These professionals face many stressors, some of which are exposure to infection, fear of infecting others, fatigue from overwork and coexistence with fatal cases, which are also linked to frustration with the feeling that it is impossible to prevent deaths. For this reason, health-promoting initiatives must be immediate and continuous, aiming to provide emotional support to professionals, helping them in the adaptation process.(3) Thus, in a recent study by the Pan American Health Organization (PAHO) in conjunction with universities in Colombia and Chile, it was revealed that, throughout Latin America, about 14.7% to 22% of the interviewed professionals presented symptoms of depressive episodes.(4)

Mostly, nursing acts in different services, from reception to intensive care. The new scenario of physical and emotional exposure during work activities presents itself to nurses at exactly the moment when the international campaign *Nursing Now* emerges to promote their empowerment as protagonists in health production. Nurses revealed the power of Nursing as a voice that comes from scientific knowledge and experience. For them, the pandemic also represents an opportunity for the profession to empower itself through ethical attitudes, continuing education and political militancy, claiming its appreciation in the face of class entities and councils.(5)

This conjuncture imposes that nursing professionals working in the hospital area are challenged daily to face the high risk of contamination, the possibility of transmitting the disease to third parties and family members, as well as the scarcity of Personal Protective Equipment (PPE), being that, in 2020, the Federal Council of Nursing (COFEN) received approximately 3.6 thousand complaints about the lack, scarcity or poor quality of PPE.(6) In addition, one can mention the creation of many beds in a short period of time, the lack of training and the adaptation to the new care with the management of patients and staff. All this, together with the historical demands of the category that still perpetuate, such as the working conditions offered, the excessive workload, the sizing and lack of personnel, the remuneration, and, until then, the social visibility (7), which, despite having been appreciated in 2020, there is much to advance.

In addition, nursing professionals are faced with the ambivalence of what would be correct and incorrect in the care provided during the pandemic, where there are moments when it is necessary to decide who will die or live, due to the high number of hospitalizations and the lack of beds.(8) Furthermore, all these feelings and uncertainties are taken outside the hospital area, in their daily lives, the frustrations, anxieties, doubts and fears continue and are accentuated by the fact that people do not comply with the worldwide recommendations for preventing the disease, so that there is, at least, a reduction in the number of cases. Considering the above, it is of interest to know if the nursing professionals who experience this reality in their work process receive attention, are cared for in terms of their anxieties, fears and uncertainties, and are being heard and welcomed. These are questions that translate the justification for this study. Accordingly, it was proposed to create a space for care during the research using the Nursing Process (NP) methodology linked to Freire’s Research Itinerary.

NP consists of a tool that enables nurses to put their knowledge into practice in an organized and qualified manner, constituting a methodological instrument, which is defined in five interrelated, interdependent and recurring steps, as follows: 1) Nursing data collection or Nursing History; 2) Nursing Diagnosis; 3) Nursing Planning; 4) Implementation; and 5) Nursing Assessment. (9) With this in mind, the objective of this study is to make a dialog about the nursing professionals’ perception regarding how they cope with COVID-19 and the repercussions on their practice and personal life.

## Methods

This is a qualitative study, typified as participatory action research, which was carried out through the Culture Circle (CC), whose theoretical construct was developed by Paulo Freire. During the study, it went through the steps of CC structured in the so-called research itinerary, as follows: 1) Generating Themes; 2) Coding and Decoding; and 3) Critical Unveiling.(10) At each step of the research itinerary, it was proposed to perform an alignment with the steps of NP, precisely to expose the care that was being offered to nursing professionals during CC. CCs are the spaces where meetings take place, where a group of people, with similar interests, discuss, dialog and reflect about their problems and life situations, based on the sharing of their experiences.(10)

In general, it is developed in person, but, in view of the need for social distance, due to the COVID-19 pandemic, it was decided to carry it out in a virtual way, through the Cisco Webex® online application, which enabled the active and simultaneous participation of nursing professionals who were invited to participate. In order to organize the Virtual Culture Circle (VCC), the researchers made an invitation via WhatsApp® available to nursing professionals from hospital institutions in Santa Catarina (Brazil). Of these, some professionals invited others within their institution, considering the Snowball sampling method.(11) Thus, the study was composed of 10 participating nursing professionals.

The inclusion criteria were nursing professionals working for one year or more in a hospital institution. VCC was held in May 2021, and the meeting time lasted a maximum of two hours, mediated by a nurse with a doctorate and experience in terms of conducting CC.

In the data collection system, methodologically, the investigation of the theme takes place initially, aiming to contemplate the first research step of the itinerary: the generating themes. This happens through a preliminary dialog where it becomes possible to identify these generating themes.(10) To that end, the following guiding question was launched: “How is it being for you to act as a nursing professional in the hospital area during the COVID-19 pandemic?”. With the question launched, the participants were instructed to access the link of the Mentimeter® online application and respond to the question with meaningful words. From this dynamic in which the generating themes were chosen, the syntheses that guided the discussion emerged: “The challenges of being a nursing professional in the pandemic”, “The learning and growth that the challenges of the pandemic have generated” and "Nursing as the protagonist of care”. In alignment with the steps of NP, the ideas that represent the generating themes correspond to the moment when data collection is held.

**Figure 1:**
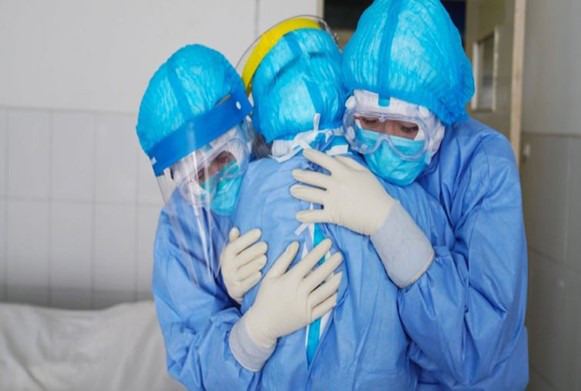
Image selected by the participants through an internet search during data collection, giving meaning to their perceptions about their professional activities during the pandemic. Photo 1. Nurse’s Day (13)

Following the data collection systematics, the second step of the research itinerary was reached. Thus, when coding, the generating themes were discussed and contextualized with the participants and, from that, they began to gain meaning, i.e., the magical view was replaced by a critical and social view of the discussed theme. This action of making the initial vision more critical, sustained the decoding, that is, at this step, even if unconsciously, an analysis of the experienced situation. This coding and decoding movement makes participants start to admire and reflect on their actions, and perceive their ability to self-transform their world and overcome the limits imposed on their daily lives.(10) In the interface with NP, there is its second step, the Nursing Diagnosis, understanding coding codes as clinical signs and decoding as the clinical judgment made considering the clinical relationships of signs and symptoms expressed during the coding process. Subsequently, still in the second step of the itinerary, the participants were encouraged to give meaning to the moment through an image that conveyed the exposed feelings. From this, the auxiliary researchers went online in search of images with the listed ideas, which resulted in five images, and one of them was selected by the nursing professionals to represent them (Figure 1 presented in the results).

In the last step of application of the research itinerary, entering the Critical Unveiling, already touched by the reflections and the meaning of the image, the mediator posed the following questions: what has been learned from this? What can you take back to your professional life? What is it like to be a nurse after what you have experienced? How has what you experienced touched you and changed you? This last step made the participants reflect on their reality and see the possibilities and the positive points in the problems they experienced.(10) In the analogy with NP, the Critical Unveiling is the moment in which care is planned and then implemented, that is, respectively the third and fourth steps of NP.

As far as data analysis is concerned, the transcription of the participants’ speeches and the analysis of the generating themes were carried out concomitantly with the development of VCC, together with the participants, since the Research Itinerary foresees an analytical process that must happen continuously and with the participation of everyone involved.(10) As a final act, the mediator invited the group to express feelings about the participation in VCC, which represents the fifth step of NP, the assessment. This was developed with the participants about the results obtained in the space for care, considering the research proposal, which was to promote care to nursing professionals. Then, the video of a song called “Laços - Homenagem aos profissionais da saúde”, composed by Gabriel Moura and performed by Nando Reis and Ana Vilela, available on the YouTube® platform, was played as a way of honoring and thanking the important work done by these nursing professionals. The dialogs of VCC were recorded and transcribed; and, for the analysis, the dialog originated in the generating themes was read and reread. It is also explained that the participants’ speeches were identified by referring to nursing theorists, aiming to honor these professionals who contributed to the structuring of science in nursing. Accordingly, the following codenames were used: Florence Nightingale, Anna Nery, Wanda Horta, Dorothea Orem, Lydia Hall, Mother Marie Domineuc, Olga Verderese, Imogene King, Edith de Magalhães Fraenkel and Mary Seacole.(12)

The research was approved by the Ethics Committee, with opinion number 4.068.387 in 2020. The Free and Informed Consent Form (FICF) was sent via Google Forms® on the day of VCC, and the participants’ anonymity was preserved by codenames as already mentioned. 

## Results

The participants were women, ages ranging from 29 to 47 years, with 1 to 17 years of experience in the hospital area, 7 working in public institutions and 3 in private ones. Of these, 3 worked in the COVID-19 ICU and the others can be seen in Table 1.

**Table 1:** Participants’ characteristics

Name	Age	Type of institution	Years working in the hospital environment	Hospital facility
Florence Nightingale	42	Public	17	Maternity and Clinical Neonatology
Anna Nery	35	Private	14	Hemodynamics
Wanda Horta	29	Public	2.5	COVID-19 Infirmary
Dorothea Orem	42	Public	7	Nursing Audit
Lydia Hall	30	Private	6	Emergency/First-Aid Room
Madre Marie Domineuc	31	Public	7.25	COVID-19 ICU
Olga Verderese	30	Public	1.25	COVID-19 ICU
Imogene King	47	Public	12	COVID-19 ICU
Edith de Magalhães Fraenkel	32	Private	5	Emergency/First-Aid Room
Mary Seacole	39	Public	10	General ICU

Following the presentation of the sociodemographic profile of the participants, the results from the data collection related to the steps of the research itinerary are presented.

Thus, the production of the generating themes triggered from the guiding question described in the method generated a “cloud of ideas”, expressed in words organized in Table 2.

This cloud of ideas allowed the researchers to access the second step of the research itinerary, that is, the coding and decoding. Accordingly, the discussion among the participants allowed to codify the generating themes and, in the deepening of this discussion, to decode them. This process is also demonstrated in Table 2.

**Table 2:** Generating themes, coding and decoding

Generating themes	Coding	Decoding
Challenging	The pandemic generated uncertainty, exhaustion, losses, anguish and fear.	The challenges of being a nursing professional in the pandemic.
Challenge		
Learning		
Knowledge		
Fear		
Adaptation		
Gratifying		
Uncertainty		
Caution	The pandemic provided the opportunity for resilience, teamwork, appreciation of the dressing, development of emotional control, overcoming, altruism and the will to learn.	The learning and growth that the challenges of the pandemic have generated.
Resilience		
Appreciation		
Study		
Courage		
Reassess		
Altruism		
Overcoming		
Team		
Exhaustion		
Growth	The pandemic provided the appreciation of nursing, the union among professionals, the organization of work, the feeling of courage and strength, professional gratitude and empowerment.	Nursing as the protagonist of care.
Organization		
Strength		
Anguish		
Emotional health		
Focus		
Dressing		
Emotional control		
Losses		
Fatigue		
Union		
Knowledge		
Fear		
Adaptation		
Gratifying		
Uncertainty		
Caution		
Resilience		
Appreciation		
Study		
Courage		
Reassess		
Altruism		
Overcoming		
Team		
Exhaustion		
Growth		
Organization		
Strength		
Anguish		
Emotional health		
Focus		
Dressing		
Emotional control		
Losses		
Fatigue		
Union		

The following are the speeches of the participants that express the syntheses established in the walk through the itinerary involving the first and second steps of this research itinerary.

### The challenges of being a nursing professional in the pandemic

*I think that, for everyone, for all the health areas involved, it was hard, in the emotional sense (*Florence Nightingale*); It really gets to a point of exhaustion, I think it’s good that it decreased, the teams were already very exhausted, we perceived that we couldn’t hold it, we couldn’t deal with so much sadness, so much loss* (Anna Nery)*; Therefore, it also shakes us emotionally, it’s heavy, it’s hard because we are not trained for these moments of loss, of mourning, these more [...] challenging moments* (Wanda Horta)*; There were several deaths during the day, the gratitude of being there, with your family well, of being able to be healthy, of being able to develop more, this was very strong for me* (Dorothea Orem); *Uncertainty, because it was an uncertain disease, there were uncertain treatments, the uncertainty of how my shift will be today* (Lydia Hall).

### The learning and growth that the challenges of the pandemic have generated

*To do our best in the beginning, so that, after that, we could reorganize the service, make care flows, and also encourage the teams, so I think it was a learning process from several points of view, technical and scientific* (Florence Nightingale)*; Learning because not only negative things came with the COVID, since we started to learn more, to seek more, in order to see what would be the best way of serving these patients* (Mother Marie Domineuc)*; We needed to go study, go after, know ourselves again, reassess ourselves, assess the team* (Lydia Hall)*; I made an effort to manage, I think that there is no professional that, at this moment, did not test, know or overcome his/her limits* (Olga Verderese)*; Therefore, we had to learn very quickly and adapt nursing care to everything we learned in college during our practice, clinical technique* (Florence Nightingale)*; We’re able to work as a team, each one with his/her own field of action, physiotherapist, psychologist, nutritionist, intensivist and nurse, so I think we strengthened the roles and competences a lot* (Mother Marie Domineuc)*; The union of the multidisciplinary team is a remarkable thing, I didn’t see this before the COVID in an intra-hospital environment* (Anna Nery)*.*

### Nursing as the protagonist of care

*We have been empowered in our profession with this pandemic* (Florence Nightingale)*; We think about reorganizing the sector, reorganizing the hospital, so I think our vision goes far beyond, we can empower ourselves in our role as nurses* (Florence Nightingale)*; The nurse has certainly been strengthened as a manager because, all the time, it required a new adaptation, a new planning, from scientific knowledge to assistance and management* (Anna Nery); *Nursing as the protagonist of care, the leadership, because we’re leaders of this care* (Florence Nightingale); *Nursing is a science that thinks about care, that plans care and that makes a whole movement of a multidisciplinary team* (Florence Nightingale); *Everyone working as equals was a difficulty and today I see that this has strengthened a lot, each one knowing the value of each profession and nursing as never before* (Anna Nery).

Moreover, in the presentation of the results, the evidence of the third step of the research itinerary is shown, the critical unveiling. In this step, the participants expressed the care developed in this period of the pandemic as differentiated, and the speech below demonstrates these perceptions.

*I think altruism is that unselfish care, not selfish, at that moment I was as a nurse, I was exercising care as a profession, but I also needed to go beyond* (Wanda Horta); *Then, when there was this second wave that was now this year, it was an even greater challenge, of various uncertainties too, a lot of strength, empathy, altruism (*Lydia Hall)

Finally, the participants’ speeches that reveal the moment of assessment of VCC are highlighted:

*I found it very welcoming and I identified with several of the speeches* (Mother Marie Domineuc)*; I think these moments are unique, because we end up in our daily routine and, within our institution, we don’t have time for this, and it was nice to hear the other fellows* (Florence Nightingale)*; It was a very welcoming moment, a chat, I would even say a refreshment for us* (Olga Verderese)*; I think these moments really strengthen us as professionals and as a profession. (*Anna Nery).

The following image is the expression of the meaning of the pandemic in practice and in the personal lives of nursing professionals, who, when participating in VCC, were able to talk and make a dialog about their perceptions. As described in the method, this is the result of an internet search for a better representation of this moment. When visualizing nursing professionals dressed and embracing, the participants selected this image as representative of their experiences in the pandemic period. 

## Discussion

As the researchers went through the phases of Freire's Research Itinerary, they correlated them with the steps of the Nursing Process (NP) based on the assumption that, in action research, one intervenes in the health reality of a given population or individual, just as nurses do when developing NP. The literature points out the relevance of using this research method whose object of study is NP. Thus, in an action research proposal in which the objective was to give new meaning to the knowledge of nurses from a hospital about NP, the importance of using this approach, in terms of its interventional character, was evidenced in the researched scenario. Firstly, it was shown how the strategy favored the detection of difficulties related to the application of NP in care practice and, subsequently, contributed to the development of the participants’ knowledge and skills in relation to NP, aiming to optimize its implementation in the health institution.(14)

Accordingly, when combining the investigation of Generating Themes with the first step of NP, i.e., data collection, the interface with the second step of NP is followed through decoding. In this sense, it should highlight the Nursing Diagnoses (ND) whose clinical judgment allowed to list from the evidence that was organized by considering the coding and decoding procedures of VCC. In the same way that in the decoding step it is possible to reflect on the situation presented and how it is theoretically supported, leading to a broadening of understanding and the elaboration of a synthesis of ideas, in the step of clinical judgment (referring to the step of NP related to diagnosis identification), an analysis is made of the real health conditions that are being assessed and, from these characteristics, the clinical analysis sustained on scientific bases is undertaken, which allows materializing the diagnostic idea.(10,15) Therefore, “the challenges of being a nursing professional in the pandemic”, one of the decoding steps worked on, were translated from the following diagnostic statement extracted from the taxonomy *North American Nursing Diagnosis Association International* (NANDA-I): 1) Stress overload related to repeated stressors characterized by impaired emotional health, negative impact of losses, exhaustion, insecurity. From a conceptual point of view, this diagnosis has the following meaning: Excessive amounts and types of demands that require action.(15)

Nevertheless, outlining considerations about the identification of this ND among the group of nurses participating in the research, which demonstrates their stress overload experienced in the face of the pandemic, it is conjectured that this stress overload in an early form was perceived in the daily lives of health professionals, especially nurses, which consists of the largest quantity of work force in this field of social action.(5) In this sense, it is discussed that anxiety, fear and insecurity surround events of this nature, and it is factual that, historically, this reality has already been experienced in similar situations. Therefore, considering the unique repercussion of this pandemic, one can revisit scientific productions whose evidence points to the psychological support to be offered in these health contexts.(16) Worldwide mental health proposals are designed to help this group of professionals who are vulnerable.(16) in this perspective, it is mentioned relevant actions of the Brazilian Ministry of Health structured based on Telemedicine and Telehealth resources, through which a teleconsultation channel is available for COVID-19 (TeleSUS) and the psychological teleconsultation service (TelePsi) for health professionals who work in the care of patients with coronavirus.(17) Furthermore, psychological support has been offered, involving psycho-educational actions by means of informative materials in physical form, or even online, using various communication platforms, as well as the services of volunteer psychology professionals, either online or in person, in university hospitals in many regions of Brazil.(18) Thus, the space for dialog that was provided to the nursing professionals participating in the study in VCC allowed the exposure of their concerns regarding the work activity around the pandemic, enabling relief from stressors by considering that, in conducting groups, communication, exchange and sharing help therapeutically_._(5)

As for the decoding: “The learning and growth that the challenges of the pandemic have generated”, it was judged that this health phenomenon is conveyed by the idea conceived in the NANDA-I diagnosis: 2) Disposition for improved coping characterized by the search for overcoming through study, ability to focus, adapt, reassess and being resilient. In NANDA-I, the concept of this ND is: pattern of valid appraisal of stressors, with cognitive and/or behavioral efforts, to control demands related to well-being, which can be improved.(19) It is debated that personal and professional growth in the work processes confers a desirable status regarding the development of skills that are required for a profession. In this sense, resilience is the necessary attribute that enables the achievement of adaptations, reformulations and re-significations in the work context. The participants of this study recognized the difficulties they called challenges and processed them aiming at the best result from the new demands.(19) It is consensual that, when there are adversities in the world of work, in order to face them healthily, it is fundamental for the emotional balance to be equalized to exercise resilience,(20) which was a noticeable movement mentioned in the research in question.

Finally, the decoding that extols “Nursing as the protagonist of care” presented characteristics that allowed diagnosing based on NANDA-I for the study participants: 3) Disposition for improved self-concept characterized by self-confidence in professional abilities and satisfaction with the sense of appreciation. In the taxonomy in question, this means the pattern of perceptions or ideas about oneself that can be improved.(19) The appreciation of nursing was never more evident than during the pandemic, where nurses were agile in making decisions based on scientific evidence and global recommendations for the restructuring of services, so that it was possible to meet the new health demand. Undeniably, they played a leading role in the situation of COVID-19 in all dimensions of care, assistance, management, politics, research and education. They composed work committees, planned the operation and physical structures to provide care, people management, the creation of protocols and care flows, and also acted directly in the care process.(21) In this vein, from the defining characteristics that were inscribed in the identified diagnosis showing the willingness to improve the self-concept of the professionals participating in the research, it was clear how much they felt appreciated in their knowledge and performance in the pandemic context in their workplace.

In the sequence, considering the phases of the Research Itinerary, the critical unveiling follows when researchers and participants dialogically construct propositions from the expanded critical consciousness provided by the previous phases. To that end, following the interconnection established in this discussion, with the steps of NP, the 3^rd^ step is reached, that is, planning, a moment in which the nurse makes clear the desired results and the nursing interventions that have the potential to respond to the results. Therefore, considering the listed ND, the next step is the identification of nursing outcomes that are formulated in the development of clinical reasoning that takes place in the sequence of steps of NP. Thus, these should configure responses to correlated health phenomena. For such selection, the taxonomy of the Nursing Outcomes Classification (NOC) was used.(22) The chosen nursing diagnoses, 1) Stress overload, 2) Enhanced coping disposition and 3) Enhanced self-concept disposition refer to the following nursing outcome goals to be achieved: 1) Stress level, 2) Coping, 3) Self-esteem and quality of life. Given these goals, interventions were extracted from the taxonomy of the Nursing Interventions Classification (NIC),(23) thus highlighting that the following interventions emerge as possibilities of action for nursing in the face of the diagnoses identified in research group of VCC: emotional support and support group, aiming to obtain responses to health phenomena captured in the course of the Research Itinerary.

In a study carried out in the Federal District in a Primary Health Care Unit, a psychology researcher structured a group with the purpose of providing an opportunity for listening, welcoming and expressing the feelings experienced by PHCU professionals during the pandemic. The experience report reveals that three meetings were held; however, the proposal is of a continuous nature, as long as it is necessary.(24) Another experience found in the literature, developed by the Pernambuco Regional Council in partnership with the Federal Nursing Council, shows that, sensitized by the situation of nursing workers, they created the Nursing Care Network, a service that provides free psychological care remotely and guidance for integrative and complementary practices in health, with the potential to help reduce stress and overcome the numerous challenges that trigger suffering of all kinds.(25)

From this perspective, involved as they were in the dialog group provided by VCC, which promoted emotional support, the participants understand that one of the great meanings of the pandemic was to allow the professional to develop a nursing care permeated by attributes such as empathy, love and solidarity, and also made them think again about the purpose of the profession, rediscovering the pleasure in being a health professional in the field of nursing, because it is gratifying to take care of human life. These feelings are uniquely reflected in their choice of image, which makes clear the strength, unity and affection in the face of the pandemic.

In this context, it is problematized that the health crisis brings ethical and humanitarian principles to the surface, thus enabling the discussion of the conceptions imbricated in the National Humanization Policy (NHP). The participants of this study extol attributes of NHP, instigated at the core of the scenario they experience. It is agreed that in situations of conflict character, whose scope is social, in this case worldwide, humanization principles are indispensable, walking towards the union of forces. Therefore, the establishment of a model of comprehensive care, based on an inter and multidisciplinary praxis should be strengthened in all levels of complexity of health care, resulting in a decrease of the biopsychosocial impact on individuals and communities.(26) When finishing the assessment of the activity, they demonstrate that the results were achieved by expressing the opportunity as a welcoming one that allows exchanges and strengthening.

As limitations of this study, it is presented that VCC used as a space for care combined with NP proved to be promising, but, despite the positive perception, it is relevant to highlight that the study reached a small number of professionals who were reached with a nursing intervention whose character was resolute, i.e., the formation of the group itself and the achieved emotional support. Naturally, when such an approach is proposed to larger groups, there is a risk of losing the welcoming essence, and it is because of this that it is important to replicate these support groups aiming to broaden their therapeutic scope.

It is concluded that VCC reveals itself as a research and learning tool configured as a space, where, despite the limitations, it provided a social interaction among the participants, with mutual exchange of experiences, with many reflections, besides expressions of feelings, experiences and learning obtained during the COVID-19 pandemic. The participants felt welcomed during the time of VCC and had positive feedbacks. The interface developed between the steps of Paulo Freire’s Research Itinerary with the steps of NP strengthened the initiative to create spaces for welcoming and listening, aiming at emotional support for nursing professionals. The application of NP made it possible to know the reality experienced by nurses working in the pandemic, making them express their perceptions that were captured as health phenomena, i.e., the defining characteristics of the selected nursing diagnoses, as well as the selection of outcomes and interventions. This dynamic attached to an action research allowed the researchers to jointly research and intervene, reaching the goals set in the application of the steps of NP, thus making it possible to visualize the application of all steps of NP.

Despite the fact that this moment highlights and appreciates the profession, nursing professionals are overloaded and exhausted by the COVID-19 pandemic, with repercussions on professional and personal life. The care for those who care needs to be planned and implemented in different scenarios, and NP built based on theoretical and scientific knowledge guide the effective improvement of the quality of health care.
